# Functional Genomics of Axons and Synapses to Understand Neurodegenerative Diseases

**DOI:** 10.3389/fncel.2021.686722

**Published:** 2021-06-25

**Authors:** Andres Di Paolo, Joaquin Garat, Guillermo Eastman, Joaquina Farias, Federico Dajas-Bailador, Pablo Smircich, José Roberto Sotelo-Silveira

**Affiliations:** ^1^Departamento de Genómica, Instituto de Investigaciones Biológicas Clemente Estable (IIBCE), Montevideo, Uruguay; ^2^Departamento de Proteínas y Ácidos Nucleicos, Instituto de Investigaciones Biológicas Clemente Estable (IIBCE), Montevideo, Uruguay; ^3^Polo de Desarrollo Universitario “Espacio de Biología Vegetal del Noreste”, Centro Universitario Regional Noreste, Universidad de la República (UdelaR), Tacuarembó, Uruguay; ^4^School of Life Sciences, Medical School Building, University of Nottingham, Nottingham, United Kingdom; ^5^Laboratorio de Interacciones Moleculares, Facultad de Ciencias, Universidad de la República (UdelaR), Montevideo, Uruguay; ^6^Departamento de Biología Celular y Molecular, Facultad de Ciencias, Universidad de la República (UdelaR), Montevideo, Uruguay

**Keywords:** axon, presynaptic compartment, transcriptomics, translatomics, proteomics, axopathologies, synaptopathies, neurodegenerative diseases

## Abstract

Functional genomics studies through transcriptomics, translatomics and proteomics have become increasingly important tools to understand the molecular basis of biological systems in the last decade. In most cases, when these approaches are applied to the nervous system, they are centered in cell bodies or somatodendritic compartments, as these are easier to isolate and, at least *in vitro*, contain most of the mRNA and proteins present in all neuronal compartments. However, key functional processes and many neuronal disorders are initiated by changes occurring far away from cell bodies, particularly in axons (axopathologies) and synapses (synaptopathies). Both neuronal compartments contain specific RNAs and proteins, which are known to vary depending on their anatomical distribution, developmental stage and function, and thus form the complex network of molecular pathways required for neuron connectivity. Modifications in these components due to metabolic, environmental, and/or genetic issues could trigger or exacerbate a neuronal disease. For this reason, detailed profiling and functional understanding of the precise changes in these compartments may thus yield new insights into the still intractable molecular basis of most neuronal disorders. In the case of synaptic dysfunctions or synaptopathies, they contribute to dozens of diseases in the human brain including neurodevelopmental (i.e., autism, Down syndrome, and epilepsy) as well as neurodegenerative disorders (i.e., Alzheimer’s and Parkinson’s diseases). Histological, biochemical, cellular, and general molecular biology techniques have been key in understanding these pathologies. Now, the growing number of omics approaches can add significant extra information at a high and wide resolution level and, used effectively, can lead to novel and insightful interpretations of the biological processes at play. This review describes current approaches that use transcriptomics, translatomics and proteomic related methods to analyze the axon and presynaptic elements, focusing on the relationship that axon and synapses have with neurodegenerative diseases.

## Introduction

Neurons are considered the most diverse class of cells known in nature (Terenzio et al., 2017), having specialized and polarized morphologies where at least five compartments can be found: cell body, dendrites, axon, presynaptic and postsynaptic domains. In addition, and although in the extracellular space, the narrow space between the pre and postsynapses defines the synaptic cleft which is also focus of major interest in order to understand the plasticity process involved in neuronal communications (Burch et al., 2017).

Particularly in the case of axons, the most evident feature is their length, which in mammal neurons could be up to five orders of magnitude larger than cell body diameters (Terenzio et al., 2017). This extraordinary characteristic requires specific and highly specialized molecular mechanisms to maintain functionality and communication with the cell body and its surrounding environment. It is known that the passive diffusion transport, which has a distance range limited to tens of micrometers at most (Kholodenko, 2003; Wiegert et al., 2007; Maday et al., 2014), and the fast and slow transport -which are the axonal main supply mechanisms- are not sufficient to ensure communication and maintenance in regions far away from cell bodies (Alvarez and Torres, 1985). Instead, pioneering studies (Koenig, 1967; Giuditta et al., 1968) and subsequent ones to the present have confirmed that axonal local protein synthesis (LPS) (Tobias and Koenig, 1975; Bassell et al., 1998; Twiss et al., 2000; Zheng et al., 2001; Verma et al., 2005; Willis et al., 2005; Shigeoka et al., 2016; Hafner et al., 2019) and glia to axon RNA and protein transfer (Lasek et al., 1977; Buchheit and Tytell, 1992; Sheller et al., 1995; Court et al., 2008; Lopez-Verrilli and Court, 2012; Sotelo et al., 2013, 2014; Canclini et al., 2020) are additional mechanisms relevant for axon growth (Campbell and Holt, 2001; Willis et al., 2007), injury response (Leung et al., 2006; Donnelly et al., 2010; Sotelo et al., 2013) and axonal regeneration (Zheng et al., 2001; Verma et al., 2005; Willis et al., 2005; Gumy et al., 2010; Obara et al., 2012; Twiss et al., 2016; Terenzio et al., 2018). Despite the current acceptance of LPS in the axon as a fundamental process in nervous system development and function, this was not the predominant paradigm in the past decades. In particular, the difficulties in identifying polyribosomes in axons by electron microscopy and other necessary elements for translation (Lasek et al., 1973) created the vision that axons can only transport molecules from/to the cell bodies and that axonal LPS was a residual phenomenon, probably ascribed to mitochondrial metabolism. Initial hints of axonal LPS came from the analysis of acetylcholinesterase protein reposition in distal mammal axonal regions, showing that axonal transport from the cell body cannot be the only axonal protein source (Koenig and Koelle, 1960). Subsequent data further supported this model (Koenig, 1967) together with evidences of LPS in other systems including the squid giant axon (Giuditta et al., 1968), the goldfish Mauthner fiber (Edström and Sjöstrand, 1969) and later in rat myelinated axons (Koenig, 1991). With the advent of modern molecular biology tools, axonal LPS was demonstrated at dozens of neuronal models during injury and/or regenerative stages. In the last two decades and especially in the last 10 years great advances were made in this field, increasing our understanding of the mechanisms involved and their importance for the development of neuronal disorders. For recent revisions about axonal LPS, please refer to Sotelo Silveira and Holt special issue (Sotelo-Silveira and Holt, 2014), and the revisions of Holt, Martin, and Schuman (Holt et al., 2019) and Twiss group (Dalla Costa et al., 2021). For a compilation about the current advances in LPS and its effects on the development of neuronal disorders, please see Lin et al. (2020) and Fernandopulle et al. (2021). Also, in the last decade have emerged functional genomics approaches that have dabbled into the study of mRNA localization and translation in the axonal territory, generating the first series of system biology pictures for this compartment (Farias et al., 2019).

In contrast, early studies in dendrites managed to identify both ribosomal particles (Bodian, 1965) and then polyribosomes (Steward and Levy, 1982; Steward and Falk, 1986), which led to the early adoption of LPS as a functional process in this neuronal compartment, hence also an early focus in its characterization (Steward and Schuman, 2001). For a recent revision about LPS in dendrites, see Chesnokova and Kolosov (2017) and Rangaraju et al. (2017). Lagging behind, recent work using transcriptomics, translatomics and proteomics tools started to shed light on the molecular components present in presynapses and the importance and complexity of axonal/presynaptic LPS (Hafner et al., 2019). However, the detailed understanding of all elements present in the presynapses, the mechanisms controlling mRNA transport, stability, protein synthesis and its regulation in the axonal/presynaptic compartment remains limited.

The recent development of sensitive and sophisticated omics protocols for the study of the nervous system dramatically increased the knowledge about the RNAs and proteins present at subcellular resolution (Koppel and Fainzilber, 2018). We are now beginning to understand the functional consequences of localizing mRNAs, ncRNAs and protein synthesis at different compartments (Farias et al., 2019) and their direct relationship to the origin and/or development of neuronal disorders. Indeed, although many neuronal disorders have at least an axon (axopathologies) or synaptic (synaptopathies) related component, the detailed molecular mechanisms involved are still largely unknown. The present review describes an overview of the methods developed to isolate axons and synaptic elements compatible with omics approaches and place these current omics advances in the context of axonal/presynaptic compartments and their relation to neurodegenerative diseases.

### Strategies to Isolate Axonal or Synaptic Material Compatible With Functional Genomics Protocols

#### Axon Isolation Strategies

**FIGURE 1:**
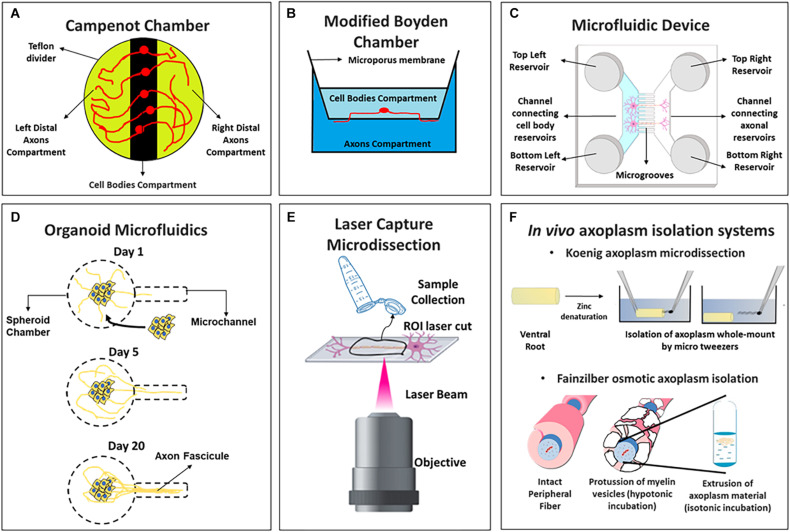
Schemes of axoplasm isolation protocols compatible with omic approaches including **(A)** Campenot chambers, **(B)** Boyden modified chambers, **(C)** microfluidic device of two channels, **(D)** organoid microfluidics, **(E)** laser capture microdissection, and **(F)** *in vivo* axoplasm isolation systems (above the Koenig’s microdissection and below Fainzilber’s osmotic axoplasm isolation).

Polarized morphology of primary neuron cultures is a crucial characteristic to consider at *in vitro* models. With the development of compartmentalized neuron cultures, it is now possible to obtain purified axon material *in vitro* for biochemical or omics studies. To date, most protocols for isolating axons have been developed using *in vitro* samples, and only a few have been developed to study the axoplasm *in vivo*, despite the differentiation state of the axonal material being an important factor. Although not all approaches might represent all situations, combining, where possible, all experimental models should be useful to understand axons in their adult environments. In the next subsections, we summarize axon isolation protocols and briefly describe relevant experimental cases where these were implemented for transcriptomics, translatomics and/or proteomic approaches. Figure 1 shows a schematic description of each axon isolation methodology and Table 1 presents a summary of omic studies developed from these strategies. Further discussion of relevant axonal omic studies can be found in Section “Functional Genomics Advances in Axon Biology.”

**TABLE 1:** Summary of axon isolation methods implemented for omics approaches.

Isolation method	Omic strategy	References
Campenot chambers	SAGE (1), Microarrays (2)	Andreassi et al., 2010; Natera-Naranjo et al., 2010
Modified Boyden chambers	Microarrays (3), RNAseq (4,5,6,7), RiboSeq (7), SILAC (7)	Willis et al., 2007; Minis et al., 2014; Taliaferro et al., 2016; Rotem et al., 2017; Zappulo et al., 2017
Microfluidic devices	Microarrays (8,9,10,11), RNAseq (12,13,14), BONCAT (12)	Taylor et al., 2009; Gumy et al., 2011; Baleriola et al., 2014; Saal et al., 2014; Briese et al., 2016; Bigler et al., 2017; Nijssen et al., 2018
Organoid microfluidics	RNAseq (15)	Akiyama et al., 2019
Laser capture microdissection	Microarrays (16), RNAseq (17)	Zivraj et al., 2010; Bellon et al., 2017
*In vivo* axoplasm microdissection (Koenig’s method)	RNAseq (18)	Farias et al., 2020
*In vivo* axoplasm extrusion (Fainzilber’s method)	MS/MS (19), iTRAQ (20), SCX-MS/MS (21), LFQ (22), BONCAT (22)	Michaelevski et al., 2010a, b; Rishal et al., 2010b; Di Paolo et al., 2021
*In vivo* FACS based	RNAseq (23), LC-MS/MS (23)	Poulopoulos et al., 2019
NanoBiopsy	RNAseq (24)	Tóth et al., 2018
Mechanical dissection	RNAseq (25)	Mathur et al., 2018
TRAP-based	RNAseq (26, 27)	Shigeoka et al., 2016; Ostroff et al., 2019
Retinal explants with cell bodies removed	pSILAC quantitative proteomics combined with single-pot solid-phase-enhanced sample (SP3) preparation (28)	Cagnetta et al., 2018
Rat cortical axons cultured in micro-patterned glass chips	Two-dimensional liquid chromatography-tandem mass spectrometry (2D-LC-MS/MS) analyses without and with stable isotope dimethyl labeling (29)	Chuang et al., 2018
Spatial transcriptomics	Ex-Seq (30)	Alon et al., 2021
IBioID (axon initial segment)	LC-MS/MS (31)	Hamdan et al., 2020

##### Campenot Chambers

Campenot chambers were originally designed to study neurite development in the presence of nerve growth factor (Campenot, 1977), but this was later expanded to neurons due to the possibility of isolating neurites from cell bodies (Campenot and MacInnis, 2004; Fantuzzo et al., 2019) (Figure 1A). An early study by Andreassi et al. (2010) used this system to report one of the first axonal transcriptomes from rat sympathetic neurons by Serial Analysis of Gene Expression (SAGE) sequencing technology. The authors identified hundreds of transcripts in axons and a specific 3′-UTR sequence in the Impa1 mRNAs that induce its NGF-dependent axonal localization and local translation. Campenot chambers were also used to profile the axonal miRNAs in sympathetic neurons, detecting 130 axonal miRNAs and reporting the enrichment of some of these (miR-15b, miR-16, miR-204, and miR-221) relative to cell bodies in distal axon regions (Natera-Naranjo et al., 2010).

##### Modified Boyden Chambers

The Boyden chamber was originally designed to study leukocyte active migration (Boyden, 1962) and was later repurposed to isolate axonal or dendritic processes by changing the membrane pore size between compartments (Figure 1B). This approach was successfully employed to study the axonal mRNA abundance in normal and injured dorsal root ganglion (DRG) axons by microarrays (Willis et al., 2007; Willis and Twiss, 2011). Subsequently, Yaron’s group combined this isolation method with RNA-Seq to report the axonal transcriptome of mouse embryo DRGs. The authors identify short sequence motifs specifically in the axonal fraction and an unexpected high proportion of mitochondria encoded mRNAs compared to those transcripts encoded by neuron soma (Minis et al., 2014). Similar approaches were used by Burge’s group to study the transcriptome of neurites from neuroblastoma (N2A) and catecholaminergic (CAD) cell lines differentiated to neurons (Taliaferro et al., 2016) while Perlson’s lab analyzed the axonal coding and non-coding RNAs from primary cultures of motor neurons in amyotrophic lateral sclerosis mutation models (Rotem et al., 2017). Finally, the group of Chekulaeva isolated neurites of mouse embryonic stem cells (mESC) differentiated to iNeurons, and performed RNA-Seq, Ribo-Seq, proteomics, pulse Stable Isotope Labeling with Amino Acids (pSILAC) and quantitative non-canonical amino acid tagging (QuanCAT) experiments to study neurite local transcriptome, translatome, total proteome and newly synthesized proteome (Zappulo et al., 2017). This report is discussed in Section “Axonal Translatomics, Proteomics and Local Synthetized Proteome.”

##### Compartmentalized Microfluidic Devices

The main advantage of this technology are the small microgrooves that separate two or more compartments allowing physical and fluidic isolation of axons from somatodendritic microenvironments *in vitro*. This system is ideal for axons or even long dendrites isolation since the length of the microgrooves can be modified and thus be used for central nervous system (CNS) axon isolation (Taylor et al., 2005; Dajas-Bailador et al., 2012; Lucci et al., 2020), which had previously not been successfully cultured in Campenot chambers. By this procedure, the Cotman lab analyzed axonal mRNA identities of mature CNS cortical neurons, reporting 300 axonal mRNAs, with many in common with those reported for peripheral nervous system (PNS) injured DRG axons (Taylor et al., 2009). A later microarray assay study the mRNA content in motor neurons in normal or a spinal muscular atrophy neuronal model (Saal et al., 2014), while the Hengst group employed RNA-Seq in rat hippocampal axons injected with Aβ_1–42_ (Baleriola et al., 2014). Both studies are further discussed in Section “Functional Genomics for the Study of Neurodegenerative Disorders in Axon and Synaptic Compartments.” More recently, the *in vitro* transcriptome of rat motor axons was reported (Briese et al., 2016), while the axonal transcriptome of distal projections was profiled in human embryonic stem cells differentiated to glutamatergic neurons (hESC-neurons) to infer human mRNA translation mechanisms (Bigler et al., 2017).

##### Organoid Microfluidics

This strategy is based on the culture of neuron spheroids which could extend a robust fascicle of axons useful for follow growth and fasciculation programs. After culturing the spheroids, neurons spontaneously generate a single, straight, and unidirectional axon fascicle within the microchannel (Kawada et al., 2017) (Figure 1D). It was employed to analyze axonal and somato-dendrite transcriptomes in isogenic human-induced pluripotent stem cells (hiPSCs)-derived motor neurons with an amyotrophic lateral sclerosis related mutation (Akiyama et al., 2019). This study will be discussed in Section “Functional Genomics for the Study of Neurodegenerative Disorders in Axon and Synaptic Compartments.”

##### Laser Capture Microdissection (LCM)

This technique consists in the isolation of regions of interest by direct observation of the sample under the microscope using a powerful laser beam and special cell culture devices (Datta et al., 2015). In the case of neurons growing *in vitro*, individual axons can be microdissected (Figure 1E). An important difficulty of this methodology is that thousands of axons need to be isolated to obtain sufficient material for further biochemical assays (Kim and Jung, 2015). Combining LCM with microarrays, Zivraj et al. (2010) studied RNA localization in axon growth cones from retinal ganglion cells (RGC) of *Xenopus laevis* and mouse at different development stages and found critical differences in mRNAs repertoire with age, suggesting that mRNA localization is important for the transformation of growth cones in mature presynaptic terminals. More recent studies use this isolation system to profile axonal miRNAs in RGC axons, identifying miR-182 as the most abundant and demonstrating its importance for axonal guidance and local translation regulation (Bellon et al., 2017).

#### Whole-Mount *in vivo* Axoplasm Isolation Protocols

Neurons from primary cultures and/or explants have metabolic differences compared to those obtained from *in vivo* tissues (Raper and Mason, 2010; Rishal et al., 2010a). Moreover, *in vivo* axons are surrounded by neuroglia and develop connections with other neurons as well, and those characteristics cannot be accurately recreated *in vitro* (Sotelo et al., 2014) -a more detailed discussion about the *in vitro* and *in vivo* implications at axonal omics can be found in sections “Axonal Transcriptomics.” Therefore, isolation of axoplasmic material *in vivo* is key to obtain information to understand axonal biology in a fully differentiated tissue. In mammals, there are two main strategies for isolating axon material from peripheral nerves *in vivo*, one derived from early work by Koenig (1965, 1986) and the other developed by Fainzilber’s lab (Hanz et al., 2003). A mechanical extrusion of axoplasm has been used as a strategy to study axons from invertebrates (Gilbert, 1972; Marquis and Webb, 1974; Morris and Lasek, 1984; Brady et al., 1985; Perlson et al., 2004) but is difficult to perform for most axons in vertebrates (Rishal et al., 2010a).

Koenig’s strategy is known as “axoplasm microdissection protocol” (Figure 1F, above) was modified to study giant axons like the goldfish Mauthner axoplasm (Koenig, 1986) but was later adapted for mammalian spinal cord ventral root axons (Koenig et al., 2000). This methodology was used to perform Real Time quantitative PCR (RT-qPCR) from rat axoplasm material (Sotelo-Silveira et al., 2007) and more recently, after modification of the protocol to increase the axoplasm’s purity, to obtain the transcriptome of mature myelinated motor axons by RNA-Seq (Farias et al., 2020).

Fainzilber’s group developed a method based on osmotic manipulations to obtain isolated axoplasm from peripheral nerves (Hanz et al., 2003; Perlson et al., 2005; Yudin et al., 2008; Michaelevski et al., 2010a; Rishal et al., 2010a) (Figure 1F, below). This method was employed to obtain the axoplasm material from sciatic nerve axons after isotonic buffer incubation (Perlson et al., 2005). By hypotonic incubations in the same model, detected 540 proteins by MS/MS with less contamination of serum proteins (Rishal et al., 2010b). Pooling from hundreds of sciatic nerve segments to reach input requirement for quantitative MS/MS combined with Isobaric Tags for Relative and Absolute Quantitation (iTRAQ), they quantified 973 proteins at injured axons (Michaelevski et al., 2010a), and nearly 900 axonal phosphoproteins (Michaelevski et al., 2010b). Our group, using the Fainzilber osmotic axoplasm extraction, recently reported the axoplasm proteome of normal and injured rat sciatic nerve with label free quantitative proteomics, identifying double number of protein groups and an axonal enrichment of ribosomal proteins relative to the whole nerve, especially at injury conditions (Di Paolo et al., 2021), showing that these methodologies are nowadays useful with state of the art quantitative mass spectrometry methods.

#### Synaptic Isolation Strategies

The detailed understanding of synapses molecular complexity has gained momentum in the last decade and the dysregulation of their components has been proposed as a contributor to several human neuronal disorders (Pocklington et al., 2006). In basic terms, a chemical synapse consists of two elements: the presynaptic and postsynaptic components. Nevertheless, it is also important to consider that most chemical synapses are in effect a “tripartite” structure, since the synaptic processes are tightly integrated with surrounding astrocytes and microglia, which are active partners in synaptic functions and relevant for plasticity related processes. For recent revisions about tripartite synapses see Heller and Rusakov (2017), Farhy-Tselnicker and Allen (2018) and for their relationship with neuronal disorders see Liu et al. (2018). It is important to highlight that although there are key implications for the glia involvement in synapses, this revision focuses on data obtained mainly on the presynaptic side of this tripartite structure.

**FIGURE 2:**
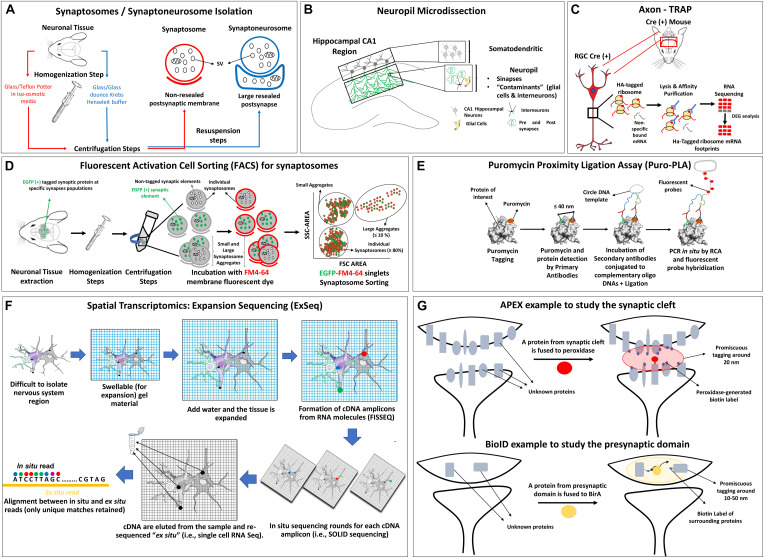
Schemes of synapse isolation protocols compatible with omic approaches including **(A)** synaptosome and synaptoneurosome, **(B)** neuropil, **(C)** axon TRAP, **(D)** FACS, **(E)** puromycin proximity ligation assay, **(F)** spatial transcriptomic example with ExSEQ protocol, **(G)** APEX and BioID protocols. SV, synaptic vesicle; HA, hemagglutinin; RGC, retinal ganglion cell; DEG, differentially expressed genes; SSC, side-scatter; FSC, forward scatter; RCA, rolling circle amplification; FISSEQ, fluorescent *in situ* sequencing of RNA.

In the next subsection we summarize synaptic isolation protocols and recent technologies to identify transcripts or proteins at subcellular resolution level compatible with omic strategies that also helps to define better the LPS capacity of the presynaptic element. In addition, the Figure 2 shows a schematic description of each synaptic isolation methodology described in the next sections and Table 2 lists omic studies which have used them.

**TABLE 2:** Summary of synaptic isolation strategies implemented for omics approaches.

Isolation method	Fraction isolated	Omic strategy	References
Centrifugation-based	Synaptosome	Microarrays (1), RNAseq (2,3, 4), RiboSeq (3), LC-MS/MS (5,6, 7), XL-MS (8)	Chang et al., 2013; Xu et al., 2013; Eshraghi et al., 2019; Noya et al., 2019; Gonzalez-Lozano et al., 2020; Gulyássy et al., 2020; Simbriger et al., 2020; Epple et al., 2021
	Synaptic vesicles	MS (9, 10)	Morciano et al., 2005; Takamori et al., 2006
Centrifugation-based + Chromatography-based purification	Synaptic vesicles	LC-MS/MS (11)	Taoufiq et al., 2020
Filter-based	Synaptoneurosome	Microarrays (12), LC-MS/MS (13)	Williams et al., 2009; Hesse et al., 2019
FACS-based	Pre or post synaptic compartment	RNAseq (14)	Arey et al., 2019
	Synaptosome	RNAseq (15), LC-MS/MS (16,17, 18)	Biesemann et al., 2014; Blumenstock et al., 2019; Hafner et al., 2019; Apóstolo et al., 2020
Neuropil Dissection	Neuropil	Microarrays (19,20), RNAseq (21), Polysome profiling (22)	Poon et al., 2006; Zhong et al., 2006; Cajigas et al., 2012; Biever et al., 2020
Immunoisolation	Synaptosomes	Immunoprecipitation, LC-MS/MS (23)	Gershoni-Emek et al., 2016
	Synaptic vesicles	ITRAQ (24,25, 26)	Grønborg et al., 2010; Boyken et al., 2013; Taoufiq et al., 2020
APEX	Synaptic cleft	ITRAQ (27)	Loh et al., 2016
iBiolD	Inhibitory post-synapsis	LC/LC-MS/MS (28)	Uezu et al., 2016
Spatial transcriptomics	Dendrites, dendritic spines, axon	Ex-Seq (29)	Alon et al., 2021

##### Synaptosomes, Synaptoneurosomes, and Neuropil Dissection

###### Synaptosomes

Synaptosomes are structures detached from neuronal projections after specific homogenization steps enriched on resealed synaptic junctions (but not resealed postsynaptic membranes) (Figure 2A, red lines). The term was first defined by Whittaker and collaborators (Whittaker et al., 1964), following previous work by de Robertis and Bennett (1955) and de Robertis et al. (1961; 1962; 1963). Synaptosomes are isolated from brain tissue homogenates in non-ionic and iso-osmotic media generally using a glass/teflon potter (Weiler, 2009). These structures retain the functional characteristics of both pre and postsynaptic sides with active membrane potential for a few hours after being extracted from live tissues, and can also be isolated from postmortem brains (Jhou and Tai, 2017; Scaduto et al., 2020). Since synapse junctions are morphologically resilient, it is possible to isolate them based on their size and density using specific homogenization and centrifugation protocols that have been extensively reviewed (Whittaker, 1993; Bai and Witzmann, 2007; Kamat et al., 2014; Michaelis et al., 2017; Wirths, 2017; Gulyássy et al., 2020). The Ficoll/sucrose (Booth and Clark, 1978) or Percoll gradients steps (Nagy et al., 1984) could be added to reduce the osmotic damage. Importantly, these methods have contaminants of axonal and glial components, and the assessment of purity is ideally based on the electron microscopy analysis of synaptosomal pellet fractions (Dunkley and Robinson, 2018). In the context of this review, we will focus on data shedding light on the capacity of the presynaptic terminal to synthesize proteins, as seen indirectly by transcriptome/translatome or directly by proteomic studies. Therefore, a technical issue where many groups are currently working on is not only gaining axonal or presynaptic insight due the unprecedented resolution/value of omic studies but also raising the bar on the quality of the samples, improving the methods to pinpoint contents of each element of the synaptic tripartite structure. This technical frontier (see sections below) is being redrawn, for example, by the purification of VGLUT1 synaptosomes with fluorescent activated cell sorting (FACS) (Luquet et al., 2017) or by spatial transcriptomics (Ståhl et al., 2016).

###### Synaptoneurosomes

Synaptoneurosomes are obtained after the homogenization of brain tissue typical with a glass/glass dounce in Krebs-Henseleit buffer, producing axonal nerve terminals (with synaptic vesicles, mitochondria, and cytoskeleton) attached to large sealed postsynaptic elements, which are almost not present in synaptosomes (Hollingsworth et al., 1985) (Figure 2A, blue lines). Synaptoneurosomes have double the size and volume compared to synaptosomes and form vesicles that appear “snowman-shaped” by electron microscopy (Villasana et al., 2006; Westmark et al., 2011; Dunkley and Robinson, 2018). A discontinuous Percoll gradient procedure was developed to isolate synaptoneurosomes in shorter times, minimizing the presence of synaptic and glial plasma membranes and of extra-synaptosomal mitochondria (Dunkley et al., 2008).

Regarding the synaptosome/synaptoneurosome contaminants and the variations on specific synaptic elements enrichment, the report of Gulyássy et al. (2020) discuss how the variations in the isolation methodologies could lead to differential results mainly due to differences in the contaminants presented in each case. This elegant meta-analysis presents five different synaptosome isolation variants and describe the source of contamination by various techniques -including electron microscopy and proteomics- and conclude that besides the core of detected proteins is the same, the differences could be important for further analysis and are a consequence mainly of the different degrees of purity from mitochondria and glial structures.

###### Neuropil dissection

This term is generically associated with specific areas in the nervous system that are composed mostly of unmyelinated axons, dendrites and glial cell processes, forming a synaptically dense region that contains a relatively low number of cell bodies (Purves et al., 2018) (Figure 2B). It can be extracted by expert microdissection, as previous reports showed for the CA1 adult rat hippocampus (Cajigas et al., 2012). The later study provided a pioneer dataset, where the power of genome-wide expression data was used to disentangle the subcellular compartment origin and possible contaminating sources. Those results were significantly improved by Hafner et al., 2019 (detailed in section “Transcriptomics and Translatomics at the Synaptic Compartments”).

It is becoming clear that the combination of approaches that use neuropil dissection/synaptosomes benefit from a combined experimental approach that uses tagging of molecular components and specific expression of tagged-translation related molecules in a time and spatial manner in order to purify contents more specifically and reducing the contamination levels.

##### Methodologies to Analyze Difficult to Isolate Synaptic Sub-compartments Compatible With Functional Genomics Protocols

Despite all the progress on the physical isolation of synaptic elements, there are certain limitations that cannot be easily resolved. For example, the presynaptic and postsynaptic compartments in the neuropil are so close from each other that it is not possible to physically isolate each part by any of the previous described protocols. Nevertheless, in the last 5 years different strategies combining sophisticated visualization methodologies with omic approaches allow to localize and identify transcripts in certain synaptic elements which cannot be easily isolated by other methodologies. One of these systems is the Axon Translating Ribosome Affinity Purification or Axon-TRAP from Holt’s lab (Shigeoka et al., 2016) to specifically analyze the axonal translatome using tagged ribosomes obtained by genetic modifications (Figure 2C). Other technology is synaptosome isolation with FACS (Figure 2D) which combines membrane fluorescent probes, cell sorting and GFP-tagging of the synaptic element of interest to isolate highly purified fluorescent synaptosomes. The method was developed by Wolf and Kapatos (1989) and adapted by Murphy lab to analyze the presynaptic transcriptome of *Caenorhabditis elegans* (Arey et al., 2019), by Macklis group to define the transcriptome and proteome of developing axonal projections of cerebral cortex (Poulopoulos et al., 2019) and by others to analyze different synaptic proteomes (Biesemann et al., 2014; Blumenstock et al., 2019; Apóstolo et al., 2020). For a general revision of FACS please see Luquet et al. (2017). In the case where the goal is to analyze LPS, the puromycin metabolic labeling of newly synthesized proteins was applied to analyze pre and postsynaptic protein synthesis combined with expansion and super resolution, and electron microscopy (Hafner et al., 2019) (Figure 2E).

To localize and identify transcripts massively in difficult-to-isolate regions, the spatial transcriptomic methods were designed, combining fluorescent *in situ* hybridization techniques with high throughput sequencing (Figure 2F). For recent revisions of these techniques and their implications on the study of the nervous system please refer to the following publications (Lein et al., 2017; Mu et al., 2019; Miedema et al., 2020; Waylen et al., 2020; Armand et al., 2021). Although not applied in depth to explore axons yet, there are variants of spatial transcriptomic protocols that were applied to study synapses. Multiplexed error-robust fluorescence *in situ* hybridization (MERFISH) (Wang et al., 2018; Xia et al., 2019) analyses hundreds of mRNA sequences at a time employing binary encoding gene labeling in multiple rounds of hybridization. Sequencing fluorescent *in situ* hybridization (SeqFISH) uses a limited pool of fluorophores to construct a temporal barcoding scheme for each round of hybridization, allowing a quantitative multiplexed single molecule mRNA imaging (Lubeck et al., 2014; Shah et al., 2016). Last, Expansion Sequencing or “ExSeq” uses fluorescent untargeted *in situ* sequencing of RNA or FISSEQ (Lee et al., 2014), combined with expansion microscopy. In essence, mRNA is retrotranscribed *in situ* to cDNA and rolling circle amplification is followed by SOLID sequencing of each cDNA amplicon using a standard fluorescent microscope. That, combined with expansion microscopy to add spatial resolution and differentiate each cDNA amplicon, is especially useful for highly packed RNA groups. As it will be discussed later, this method provides very interesting results on the local transcriptome of neuronal sub-compartment of dendrites, like spines *in vivo* (Alon et al., 2021).

In relation to *in vivo* proteomics, studies partially independent from physical isolation protocols, two methods were designed both based in the promiscuous tagging of a protein of interest and the surrounding proteins (approximately 10–50 nm around) with biotin via ascorbate, known as APEX, or modified *Escherichia coli* biotin ligase, known as BioID, which are then recovered by streptavidin or anti-biotin beads and sequenced by MS (revised by Han et al., 2018; Wang and Savas, 2018; Bosch et al., 2021) (see Figure 2G). Interactions networks with a byte localized in the synaptic cleft, can be pinpointed allowing sub-nanometer resolution of structures far away from the reach of other classical isolation procedures (Loh et al., 2016).

A detailed discussion about early and recent works that used these technologies to analyze synapses by functional genomics can be found in Section “Functional Genomics for the Study of the Synaptic Compartment.”

### Functional Genomics Advances in Axon Biology

#### Axonal Transcriptomics

Transcriptomic analysis allows the investigation of the axonal mRNA repertoire, which represents proteins that could be potentially synthesized in axons. There are almost 20 published axonal transcriptomes to date (Willis et al., 2007; Taylor et al., 2009; Andreassi et al., 2010; Zivraj et al., 2010; Baleriola et al., 2014; Minis et al., 2014; Saal et al., 2014; Briese et al., 2016; Taliaferro et al., 2016; Bigler et al., 2017; Rotem et al., 2017; Zappulo et al., 2017; Mathur et al., 2018; Tóth et al., 2018; Poulopoulos et al., 2019; Farias et al., 2020), obtained from various species and neuronal types. In addition, although many of these studies focused on mRNAs, RNA-Seq approaches have also been successfully applied to the investigation of axonal miRNAs (Phay et al., 2015; Bellon et al., 2017; Rotem et al., 2017; Corradi et al., 2020), expanding the research into regulatory mechanisms of local mRNA translation.

Most of these studies analyzed the enriched RNAs in the axon compared to the somatodendritic domain. This strategy is inherently conservative because it eliminates from the analysis those RNAs that might be present at high levels in both domains like the β-actin which localization and local synthesis in axons have been demonstrated (Wong et al., 2017). Despite this caveat, the data from these studies has provided significant progress for the field.

While most studies were carried out using *in vitro* models, data on what occurs under *in vivo* conditions has recently been obtained. The work of Macklis’s lab described the local transcriptome and proteome of growth cones from mouse cerebral cortex single projections, revealing novel molecular specializations of these structures, including accumulations of the mTOR2 and mRNAs that contain mTOR-dependent motifs (Poulopoulos et al., 2019). In addition to this, a recent study from the Sotelo-Silveira group revealed the axonal transcriptome of fully differentiated myelinated motor neurons derived from rat ventral roots (Farias et al., 2020). In this study, the population of axonal mRNAs detected showed less diversity compared with previously published transcriptomes obtained from *in vitro* cell cultures. This result may be explained by multiple factors, including differences between neuronal cell cultures *in vitro* and neurons in their normal environment, differences in the neurons’ developmental stage, and/or differences in the axonal isolation protocols. Interestingly, almost all mRNAs coding for ribosomal proteins were found in the axoplasm, providing clues to how translation machinery may renew and operate far away from the nucleolus. The latter is consistent with proteomics results indicating that ribosomes are enriched in sciatic nerve axons preparing to regenerate (Di Paolo et al., 2021).

The comparative analysis of axonal transcriptomes shows that the gene ontology categories related to translation, mitochondria, and the cytoskeleton are enriched in all data sets, regardless of neuronal type or state of maturation (Farias et al., 2019). These categories are also enriched in the group of mRNAs present in axons of different types (sensory or motor) or various differentiation conditions (mature or in active growth), defining a core set of mRNAs needed for the correct function of the domain (Farias et al., 2020). Other ontological categories are also enriched in some of the axons, such as intracellular traffic, axon guidance, and proteins related to the synapses (Farias et al., 2019). In turn, studies have also shown how the axonal transcriptome can dynamically change throughout neuronal development, varying the capacity for protein synthesis in axons at different development stages. The study of mRNAs located in growth cones of the RGC of *Xenopus* in stages of “pathfinding” and “target-arriving” revealed that the number and complexity of RNAs increased dramatically with age and adapted to the functional demands of the growing axon tip as it transforms into the presynaptic terminal (Zivraj et al., 2010). Furthermore, the examination of axonal transcriptomes from embryonic and adult stages of mouse DRG allowed the estimation that there are a similar number of localized transcripts at both stages but with significant changes in the identity of the localized mRNAs. This suggests that, although the repertoire of localized mRNAs changes, the overall ability to transport axonal mRNAs does not change from development to adulthood (Gumy et al., 2011). The later observation was supported by the trapping of ribosome associated mRNAs at different developmental (Shigeoka et al., 2016) and adulthood stages associated with learning processes in axons (Ostroff et al., 2019), see section below.

#### Axonal Translatomics, Proteomics and Local Synthetized Proteome

##### Axon Translatomics

First axon translatomics was performed by Shigeoka et al. (2016) with Axon-TRAP, a method to analyze the mRNAs associated to ribosomes targeted into axons or synapses of interest *in vivo* (Figure 2C). Approximately, 2,000 transcripts were associated with tagged ribosomes, decreasing to a half in the adult axons, showing also the first conclusive evidence of LPS in mature CNS axons (Shigeoka et al., 2016). Moreover, the comparison of the axonal translatome repertoire with the mRNAs previously reported by transcriptome assays at the same developmental stages (Zivraj et al., 2010) shows coincidences, but also shows a group of genes that are detected only at the transcriptome level, suggesting the existence of repressed mRNAs that are not expressed until a certain development stage. The authors also bring evidence of alternative splicing events, that could produce specific UTR sequences that define the mRNA transport to the axon, and suggest *cis*-regulatory elements and secondary RNA motifs in the axon-localized mRNAs that could promote axonal translation. The TRAP technology was recently applied in axons of rat adult forebrains *in vivo* and linked local translation with learning and memory related processes (Ostroff et al., 2019). Interestingly, the number of transcripts detected in the adult axon in CNS by both studies as being translated are in the range of the ones detected later in adult myelinated PNS motor axons (Farias et al., 2020), likely indicating that adult differentiated axons have less mRNA diversity (see above, “Axonal Transcriptomics”).

##### Axon Proteomics and Local Proteome Assays

The increasing capacity of mass spectrometry technologies allow the study of samples with low quantities of total proteins like the axons by proteomic approaches. The total axonal proteome was analyzed mostly at *in vitro* models and together with local synthetized proteomes to perform comparative analysis.

Zappulo et al. (2017) report the transcriptome, translatome, proteome and newly synthetized proteome of mice iNeurons neurites, identifying approximately 7000 proteins and revealing that the neurite enriched mRNAs encoded approximately half of the neurite-localized proteome in relation to cell bodies. Moreover, the Ribo-Seq neurite data indicated a preferential translation of localized RNAs and proteins in neurites with a strong correlation with the newly synthetized proteome obtained by pSILAC and QuanCAT protocols validated by puromycin proximity ligation assays. These results suggest that those localized mRNAs are indeed translated locally. Most of these proteins have neuronal related functions associated with neuronal diseases and have a relevant proportion of RNA binding proteins important for mRNA translation regulation and localization.

The study of LPS regulation at a genome wide level has only recently been explored. Cagnetta et al. (2018) combine elegant ultrasensitive sample preparation technology known as single-pot solid-phase-enhanced sample preparation (SP3) with pSILAC metabolic labeling and compartmentalized chambers to isolate the axons of RGC *X. laevis* embryos and demonstrate that axon local translation could be regulated by specific cues including BDNF, semaphorins and Netrin-1. Each cue differentially affects axonal synthesis of multiple proteins in a temporally dynamic way (Cagnetta et al., 2018). In general, this approach was able to observe a thousand newly synthetized proteins in categories related to ribosome, translation and proteosome related processes, a higher detection capacity compared to the axonal enriched proteins reported for cortical axons in culture (Chuang et al., 2018).

The approaches mentioned above cannot differentiate between specific subregions of the axonal domain, a challenge that may be overcome by promiscuous tagging protocols. In this regard a recent report adapted the BioID protocol to analyze the proteome of intracellular membrane, cytosolic, and microtubule compartments of the axon initial segment of E18 rat hippocampal neurons (Hamdan et al., 2020). The authors report an enrichment on cytoskeleton related proteins (i.e., ankyrins, spectrins, actin, and tubulins), proteins related to axonal trafficking (i.e., Mical3) and no previous axonal assigned proteins that open new questions about its roles in neuronal functions.

Most of the axonal proteomics are carried out *in vitro*, losing the influence of *in vivo* cell-to-cell interactions. Nevertheless, a few axonal proteomic analyses have been developed *in vivo* by the group of Perlson et al. (2005) and recently used by our group. The first *in vivo* axonal proteome was obtained in 2005 using isotonic buffer incubations (Perlson et al., 2005). Later, improvements in the protocol after the addition of a hypotonic buffer incubation step combined with iTRAQ proteomics allowed authors to analyze the axoplasmic proteome in injured rat sciatic nerve ligatures and identify 973 proteins (Michaelevski et al., 2010a). A publication of our group detected 2,087 proteins in extruded axoplasm from rat sciatic nerves *in vivo* with an enrichment on ribosomal proteins at injured axons and reports a list of newly synthesized proteins in the axoplasm *in vivo* obtained by BONCAT-MS/MS protocols (Di Paolo et al., 2021). Functional categories obtained from the latter are also consistent with an *in vivo* axon growth cone proteome from developing mice cortex (Poulopoulos et al., 2019).

It’s important to note that there is also the possibility of a contribution from the surrounding glia to the local axonal proteome (Court et al., 2008; Lopez-Verrilli and Court, 2012; Sotelo et al., 2013, 2014; Canclini et al., 2020), therefore, further refinement of techniques should also consider this source in upcoming experiments.

### Functional Genomics for the Study of the Synaptic Compartment

Despite all the progress made in the study of synapses at a local level, the identities of most synaptic mRNAs, ncRNAs and proteins were unknown until the advent of next generation RNA sequencing and proteomics, which coincided with the increased capacity to isolate synaptic components. As mentioned above most omic analyses focus on the postsynaptic side (Sasaki, 2020). In the next sub-sections, we summarize the current transcriptomics and proteomics advances, focusing on presynaptic studies.

#### Transcriptomics and Translatomics at the Synaptic Compartments

In the early nineties most of the studies to define the synaptic RNAs used Northern blot or RT-PCR to analyze synaptosome fractions, assuming that the presynaptic side lacked local mRNA translation (Steward, 1983) and therefore, was no need to distinguish between pre and postsynaptic regions (Steward and Banker, 1992). Nevertheless, the group of Giuditta et al. (2008) mounted solid evidence of mRNA presence and LPS in the axon and later at the presynaptic endings in several publications (see reviews from Crispino et al., 2014), including pioneer cDNA libraries from the squid giant axon (Kaplan et al., 1992) and demonstration that active polysomes were present in presynaptic endings of squid brain synaptosomes (Crispino et al., 1993, 1997; Eyman et al., 2007).

Initial microarrays studies on tissues with complex mixtures of neuronal and glial processes detected hundreds of potentially pre and postsynaptic mRNAs, but since the axon side of the story was not considered to be relevant, the studies likely miss classify axonal mRNAs as dendritic ones (Poon et al., 2006; Zhong et al., 2006). Later Zivraj et al. (2010) identified, using also microarrays and microdissection of *X. laevis* growth cones, 958 transcripts, and reported a low intersection with previous datasets suggesting that the synaptic mRNA population seems to be more complex and dynamic across maturation. In addition, other studies added the presence of miRNA inside synaptic vesicles by microarrays (Xu et al., 2013; Li et al., 2015) and more recently the small non-coding synaptic RNAome from mice hippocampal synaptosomes and suggests that synaptic microRNAs can potentially control almost all the synaptic mRNAome (Epple et al., 2021).

However, the most significant synaptic omic improvements came with next generation sequencing protocols (NGS) combined with the strategies mentioned in Section “Methodologies to Analyze Difficult to Isolate Synaptic Sub-Compartments Compatible With Functional Genomics Protocols.” Early work from Schuman’s lab performed a profile of the adult rat hippocampal neuropil transcriptome, which is highly enriched on sealed synapses with pre and postsynaptic components (Cajigas et al., 2012). The authors identified 8,379 transcripts of which 2,550 mRNAs were associated with dendritic or axonal synaptic compartments, excluding those transcripts enriched in non-neurite data sets. The mRNA population discovered suggested that local translation in mature synapses could be a commonly occurring mechanism, rather than an exception, later confirmed in detail by Hafner et al. (2019) (see below). Despite possible contaminations that are always present in neuropil preparations (discussed in Section “Synaptosomes, Synaptoneurosomes, and Neuropil Dissection”), this observation started to move the debate beyond local translation as a process relevant not only in development or regenerating axons but toward its validation in mature axons and synaptic contexts. Indeed, Axon-TRAP protocols (see above) supports this point demonstrating that local mRNA translation occurs in CNS axons and is tightly regulated during development (Shigeoka et al., 2016; Ostroff et al., 2019).

The neuropil analysis cannot distinguish between pre and postsynaptic regions which is important in order to demonstrate if LPS occur at both axonal and dendritic sides. To answer this point an exhaustive analysis from Schuman group combined FACS synaptosomes isolation procedures, puromycin-based metabolic labeling (to visualize newly synthetized proteins *in situ* by fluorescent and electron microscopy imaging), expansion and super resolution microscopy to clearly differentiate the pre and postsynaptic sides, and demonstrate the occurrence of LPS at postsynaptic but also at excitatory (VGLUT^+^) and inhibitory (vGAT^+^) presynaptic terminals (Hafner et al., 2019). The authors suggest by synaptosome transcriptomics, and confirmed by fluorescent super resolution imaging, the presence of translation machinery and ribosomes in presynaptic terminals and directly evidence local translation in these compartments by puromycin labeling combined with expansion microscopy or electron microscopy imaging. In the same year, Arey et al. (2019) designed a method based on differential fluorescent labeling of cell bodies, presynaptic and axonal regions (using specific protein markers fused to fluorescent tags) that could be isolated by FACS protocol to perform transcriptomics specifically for each compartment in *C. elegans*. The authors detected 8,778 transcripts at presynapses with 542 enriched relative to cell bodies, an overrepresentation of RNA binding proteins and translation regulation RNAs and also a recurrent PUMILIO binding motif in the 3′-UTR region that could regulate memory related processes.

Recently two synaptic translatomics assays were developed in order to have a closer picture of the local regulation of protein synthesis at synapses. Gkogkas’ lab that combined a well-established method for synaptoneurosome preparation with Ribo-Seq in order to assess translational levels of synapse-associated mRNAs, identifying transcripts from over 10,000 protein-coding genes but cannot distinguish between pre and postsynaptic sides (Simbriger et al., 2020). To assess this point, the group of Schuman recently published a method to perform Ribo-Seq on microdissected neuropil and somata from CA1 region of rat hippocampal slides to differentiate translational activity of polysome and monosome fractions (Biever et al., 2020). The authors demonstrate that neuropil extracted monosomes are active in dendrites and axons, translating synaptic related transcripts that encoded highly abundant proteins (detected by neuropil proteomics). The same group is currently working on the translatome and transcriptome profiles of the neuropil to unravel the unprecedented synaptic capacity for local protein production *in vivo* which is essential to maintain and modify the pre and postsynaptic proteome (available at bioRxiv). Is relevant to note that the active monosome fraction in axons may explain the historical difficulties to visualize polyribosomes in axons by electron microscopy imaging since monosomes are almost impossible to detect by these methodologies.

As we discussed above, the isolation of pure synaptic compartments sometimes is difficult to achieve. To overcome some of these inherent difficulties and limitations a recently developed group of techniques termed “spatial transcriptomics” may allow specific subcellular localization of individual mRNA at massive scales (see Section “Methodologies to Analyze Difficult to Isolate Synaptic Sub-Compartments Compatible With Functional Genomics Protocols”). Moffitt et al. (2018) used MERFISH combined with single cell transcriptomics to investigate the molecular, spatial, and functional organization of the mouse hypothalamic preoptic region, identifying nearly 70 different neuronal populations and demonstrating the capacity of this methodology to analyze complex brain circuits. The same group is currently attempting to molecularly define the mouse primary motor cortex transcripts with high spatial resolution and the transcriptome compositions in somata, dendrites, and axons of individual mouse hippocampal neurons (available at bioRxiv). SeqFISH was used to reveal the intra-regional complexity of dentate gyrus, CA1 and CA3 from mouse hippocampus *in vivo* showing an increased cell heterogeneity along the dorsal to ventral axis (Eng et al., 2019). Finally, Alon et al. (2021) developed the “ExSeq” protocols to reveal the RNA population of dendrites, spines and even axons from mouse hippocampal neurons, detecting differences between mRNA populations in dendrites and their own spines. With this system the authors reveal the presence of multiple RNA types (small RNAs and even intron retaining transcripts) at distal dendrites 100 microns away from the cell bodies and could also recapitulate the organization of neuron types from mouse primary visual cortex by the detection of transcripts by the ExSeq protocol. Importantly, this technology has the potential to be used to study the transcriptome of presynapses without complex isolation procedures.

Regarding the bioinformatic analysis of the data obtained from single cell sequencing protocols, and despite global similarities with standard RNA-Seq analysis, it has some specific aspects to consider. Recent revisions summarize the bioinformatic protocols that have been specially designed for these methodologies and the main aspects to be considered (Kulkarni et al., 2019).

All these recent findings have transformed the previously prevailing concept that local translation was only a minor source of synaptic protein content, and instead confirmed it not only as a mechanism used during synaptic plasticity in dendritic domains but also on the presynaptic side at basal conditions.

#### Proteomics at the Synaptic Compartments

The analysis of protein content in synaptosomes and derived sub-fractions lead to an exponential increase in our knowledge of synaptic protein content. All the intensive proteomics work performed in the last 20 years defining the current structural and functional blueprint of synapses that now support LPS studies is difficult to review in a short section, so we refer for example to Laßek et al. (2015); Dieterich and Kreutz (2016), and Xu et al. (2021). With the advent of quantitative and unbiased proteomics, it is now possible to evaluate the precise neuronal proteome changes associated with diseases (Reig-Viader et al., 2018; Wang and Savas, 2018) or plasticity related processes. The recent report from Noya et al. (2019) exemplifies how proteomics could explain synaptic plasticity functions. The authors isolate synaptoneurosomes from mouse brains and perform transcriptomics and proteomics protocols to demonstrate the role of synapses in the local control of the circadian rhythm, revealing the largest proportion of circadian transcripts in any model described to date and suggesting that synaptic protein local translation could have an essential role in sleep and wake states which are essential for regulate memory and learning efficiencies diurnally (Noya et al., 2019).

An inherent problem of synaptic proteomics is the loose of spatial information during the MS/MS analytical process. The isolation of synaptic plasma membranes or SPMs (Jones and Matus, 1974), by osmotic lysis combined with differential centrifugation, and synaptic vesicles or SVs (De Camilli et al., 1983; Huttner et al., 1983) were the initial attempts to obtain spatial information, besides the considerable contamination obtained. Immunoprofiling synaptic vesicles docked to the presynaptic active zone (Morciano et al., 2005, 2009; Takamori et al., 2006), together with a step of mild proteolysis of synaptosomes (Boyken et al., 2013), helped to increase purity, gaining insights on differences, for example between glutamatergic and GABAergic synapses (Grønborg et al., 2010). Recently, ultra-definition subcellular proteomic workflows, detected 1,500 SVs proteins (Taoufiq et al., 2020) increasing several fold previous capacity detection levels.

In many cases synaptic regions are still difficult to physically separate from other parts (i.e., the pre and postsynaptic regions of the neuropil) or almost impossible to isolate, such as those from the synaptic cleft. Recent protocols were modified to direct the expression of the “promiscuous” tagging enzymes to the subcellular region of interest. In this line the APEX protocol was applied to study the synaptic cleft from excitatory (glutamate releasing) and inhibitory (GABA releasing) synapses from adult mouse brain *in vivo*, reporting 199 glutamatergic and 42 GABAergic unique proteins, assigning previous known synaptic proteins to a specific cleft type and reporting novel synaptic proteins (Loh et al., 2016). In the same year the BioID protocol was applied to characterize the inhibitory postsynaptic (iPSD) proteome of mouse brain identifying all previous known iPSDs proteins, reporting 140 novel ones and a few protein groups were also detected in excitatory postsynapses suggesting a more complex scenario than previously thought (Uezu et al., 2016). It will be very interesting to see the result of combining APEX/BioID like approaches with methods to evidence local protein neo synthesis at proteomics scales in axon/synapses.

### Functional Genomics for the Study of Neurodegenerative Disorders in Axon and Synaptic Compartments

A large and heterogeneous group of neuronal disorders are those of the brain and spinal cord that are characterized by the progressive deterioration of neuron structures and functional capabilities. The literature of neuronal disorders due to molecular disbalances at the axonal and/or synaptic levels (known as axonopathies and synaptopathies, respectively) is vast, covering dozens of methodological approaches and having tons of relevant results on the understanding of the molecular pathways involved, and changes that could trigger each neuronal disease. Nevertheless, the ratio between advancements in basic science and the development of biomedical therapies able to cure or at least reduce the symptoms and/or progression of most neuronal disorders is depressingly low and, in some cases -like Alzheimer’s Disease or Amyotrophic Lateral Sclerosis-, there is almost no treatments to slow down the progress of the symptoms.

In general, neuronal disorders are studied based on a reduced number of their individual cellular components (Faundez et al., 2019). Nevertheless, even in monogenic neurological disorders (i.e., Rett syndrome or Huntington’s disease), the proteins affected are capable of regulating the function and/or expression of hundreds/thousands of genes. In this context, system biology approaches appear as the main way to have a comprehensive picture of the observed phenotype. Functional genomic approaches are providing previously unexplored regulatory events that need to be pursued using gene centered approaches to validate observations in the context of the pathologies studied.

For the purpose of this review, we were interested in studies where axon and/or synaptic functional genomic studies provided any new insight on the relationship of the local capacity of gene expression in these compartments and the neuronal disorders analyzed. Since there are many relevant articles in this area especially in the case of synaptopathies, where is difficult to disentangle the influence of the cellular soma from the local response, we focused on diseases such as Alzheimer’s Disease (AD), Amyotrophic Lateral Sclerosis (ALS), and Spinal Muscular Atrophy (SMA), to provide examples on how local gene expression could be of importance. Interestingly, it is commonly observed that that changes in axons and or synapses tend to occur early in the degeneration of the implicated system, highlighting the local influence but also possible venues for therapeutic development. Moreover, several reports shows that initial synaptic dysfunction and associated cognitive impairments can be reverted during early stages of neuronal disorders (Lacor, 2007; Zoghbi and Bear, 2012). In Tables 3, 4 we summarize important omics studies developed to understand these neurodegenerative diseases from axon or synaptic compartments, respectively.

**TABLE 3:** Summary of axonal omic approaches implemented for the study of neurodegenerative diseases.

Axon isolation strategy	Omic strategy	Neuronal disorder analyzed	Main discoveries	References
Rat embryonic hippocampal neurons cultured in tripartite microfluidic chambers	RNA-seq	Alzheimer’s disease (AD)	• Locally applied Aβ_1__–__42_ triggers recruitment of mRNAs into axons and local translation. • ATF4 is local translated, is necessary for retrograde spread of Aβ_1__–__42_, induce neurodegeneration *in vivo* and is increased in AD.	Baleriola et al., 2014
Mice embryo motoneurons cultured in 2 channel microfluidic device	Microarrays	Spinal muscular atrophy (SMA)	• SMN knockdown produce downregulation of synaptic localization, neuron projections and growth cones related mRNAs. • Transcripts related to local translation and energy production are enriched relative to cell bodies. • Dysregulation of transcripts in axons and cell bodies could trigger SMA.	Saal et al., 2014
Mice embryo spinal cord motoneurons cultured in Boyden modified chambers	RNA-seq (analysis of mRNAs and miRNAs)	Amyotrophic lateral sclerosis (ALS)	• SOD1G93A and TDP43A315T mutations of ALS show enrichment in mitochondria related mRNAs; most genes altered in the SOD1 model were not altered in the TDP43 model; novel list of axonal miRNAs with altered expression levels. • Open the possibility that axon local translation could module initial stages of ALS and bring possible teraphies.	Rotem et al., 2017
mESC derived motoneurons cultured in 2 channel microfluidic device	RNA-seq (axon-seq in house method)	Amyotrophic lateral sclerosis (ALS)	• Improved sensibility in axonal RNA-Seq protocols; Identification of dysregulated genes in axons of SOD1G93A mutation ALS model including previous known ALS-causing genes. • Axon seq could be a robust, sensible and of low cost RNA Sequencing for polarized cells.	Nijssen et al., 2018
Isogenic human-induced pluripotent stem cell-derived motor neurons cultured as nerve organoid in microfluidic device	RNA-seq	Amyotrophic lateral sclerosis (ALS)	• Identification of aberrant axon branching and increased levels of Fos-b mRNA. Overexpression of Fos-b produced aberrant axon branching *in vivo* and siRNA treatment ameliorated the observed phenotype. • Provide a complete RNA profile of human motor neurons, provides a novel strategy for neurodegenerative axon analysis and present Fos-b pathway as potential therapeutic target in future ALS research.	Akiyama et al., 2019

**TABLE 4:** Summary of synaptic omic approaches for the study of neurodegenerative diseases.

Synaptic isolation strategy	Omic strategy	Neuronal disorder analyzed	Main discoveries	References
Synaptoneurosomes from postmortem human prefrontal cortex	Microarrays	Incipient Alzheimer’s disease (IAD)	• Increased expression of neuroplasticity related mRNAs including GluR2 and CHRM3. • GluR could have an incidence in AD development and CHRM3 a compensatory mechanism of IAD patients – 3′UTR conserved motifs.	Williams et al., 2009
Synaptosomes from human postmortem hippocampus and temporal cortex	Proteomics	Incipient Alzheimer’s disease (IAD)	• 26 proteins involving different cellular functions (energy metabolism, signal transduction, vesicle transport, structure, and antioxidant activity) were differentially expressed between AD and control subjects involved in synaptic dysfunction.	Chang et al., 2013
Synaptoneurosome from human post mortem superior temporal gyrus and primary visual cortex	Proteomics	Alzheimer’s Disease (AD) with known APOE gene status	• Identification of 5,500 proteins in human brain synapses. • Decrease abundance of proteins important for synaptic and mitochondrial function and an increased on those involved in neuroimmune interactions and intracellular signaling at AD brain synapses.	Hesse et al., 2019
Synaptosomes from spinal cords central synapses of P14 Smn2B/- mice	Proteomics	Spinal muscular atrophy (SMA)	• 65 proteins differentially expressed at early presymptomatic stages with enriched molecular functions related to mitochondrial dynamics, cholesterol biogenesis and protein clearance. • These proteins are involved in cellular functions including energy metabolism, signal transduction, vesicle transport, structure, and antioxidant activity – uncover potential markers for pathogenic mechanism that triggers synaptic dysfunction.	Eshraghi et al., 2019
Synaptosomes from SOD1G93A mice model	Proteomics	Amyotrophic lateral sclerosis (ALS)	• Perform network analysis and identified Staufen1 as major mediator of dynein interactions trough PP1B protein. • Demonstrate that Dynein-Staufen-PP1B interactions was altered in ALS models. • Suggest a model in which dynein at synases do clustering and anchoring of mRNAs including Staufen and these interaction regulate mRNA localization along the axon and the synapses and is altereded in ALS models	Gershoni-Emek et al., 2016

#### Alzheimer’s Disease

Alzheimer’s disease (AD) is the most common type of dementia, and although some therapies can temporally ameliorate some of the symptoms (Long and Holtzman, 2019; Breijyeh and Karaman, 2020; Zetterberg and Bendlin, 2021), none can stop disease progression. AD pathogenesis is complex, involving abnormal amyloid beta (Aβ) metabolism, tau hyperphosphorylation, oxidative stress, reactive glia and microglia changes, and other pathological events [please refer to Khan and Bloom (2016); Wang et al. (2017), Femminella et al. (2018), and Guo et al. (2020)]. Accumulation of extracellular oligomeric forms of Aβ is positively correlated with the onset of cognitive decline in AD brains and can elicit neurodegeneration in primary neurons (Zhang et al., 2012; Lee et al., 2020). Elevated Aβ levels can have an effect on the axons, triggering pathogenic signaling mechanisms (Kanaan et al., 2013; Baleriola et al., 2014).

The relation between AD and axon biology is currently centered in recurrent evidences that presents axonal degeneration as a process that occurs before cell body loss (Adalbert and Coleman, 2013) and before the onset of clinical symptoms, which seems to be a common feature of age-related diseases including ALS (Dadon-Nachum et al., 2011) and Parkinson Disease (Tagliaferro and Burke, 2016). This characteristic opens the possibility of potentially reversible disease-modifying phase for these pathologies. In consequence, understanding the molecular and cellular mechanisms underlying these axonal processes is critical for the development of therapeutic strategies (Salvadores et al., 2017). Although it is now clear that changes at synapses during AD development produce clear dysfunction of synaptic activity, the mechanisms involved are still elusive. In this line, recent proteomic analysis about the physiological roles of amyloid precursor protein (APP) shows that this protein is present in the presynaptic active zone and links the progress of AD with the physiological role of APP in synaptic vesicle traffic (Laßek et al., 2013; Weingarten et al., 2017). Besides the fact that AD studies in the last 30 years have been mainly centered around Aβ plaques and tau aggregations, more recent interpretations for sporadic AD development have started to focus on the multitude of dysregulated events that eventually impact synapse function and lead to neuronal loss (Sheng et al., 2012). Indeed, a recent revision from Jackson et al. (2019) describe the relative direct and indirect effects of Aβ and tau aggregations on synaptic dysfunction.

It is interesting to note that most of the functional genomics data developed in AD models does not discern between the local effects at neuronal compartments from the total differences at each brain region where cell bodies and other non-neuronal cells are prevalent than axon/dendrite contributions. Nevertheless, this type of omics approaches provides data about the synaptic roles in AD progression. For example, the study of Kempf et al. (2016) analyzed different brain regions from APPSwe/PS1ΔE9 mouse, a widely used model of cerebral amyloids, and reveals differential molecular signatures on synaptic plasticity related pathways for each brain region. More recently, membrane-enriched post-mortem brain samples (including synaptic elements) were analyzed by label free quantitative proteomics in free of pathology, asymptomatic and AD patients. A total of 530 unique proteins with significantly altered expression levels were observed across each condition, but only two proteins (APP and SNAP25) changing between all groups: APP increases and SNAP25 decreases throughout AD (Higginbotham et al., 2019).

Regarding the functional genomics studies performed at synaptic or axonal compartments locally, Miller’s group isolated synaptoneurosomes from the prefrontal cortex of control and early AD patients and performed microarray analysis (Williams et al., 2009). The authors found increased expression of neuroplasticity related genes, UTR consensus sequences related to translation regulation at synapses and several neurotransmitter receptors, including the GluR2 subunit of the AMPA receptor and the muscarinic cholinergic receptor 3 (CHRM3). Few years later Yang et al. (2013) perform one of the first shotgun proteomic assay in whole brains from early AD stage using a TBA42 mutant and wild type mice. They found 3 differentially expressed proteins and suggesting early signatures of imminent neurodegeneration long before behavioral changes appear (Yang et al., 2013). Other independent study performed proteomics on isolated synaptosomes from human brain autopsies with AD and respective controls, reporting 26 differential synaptic proteins with functions related to energy metabolism, signal transduction, vesicle transport, structure, and antioxidant activity (Chang et al., 2013). In addition, a recent work analyzes the synapse disruption due to Aβ aggregates and perform an unbiased proteomic screen plus a systematic *in silico* analysis of synaptoneurosome preparations from human AD brain cortices with a known APOE genotype variants and controls subjects (Hesse et al., 2019). The authors detected 5500 proteins with a decrease of proteins relevant for synaptic and mitochondrial function and an increase on those involved in neuroimmune interactions and intracellular signaling.

Most of the previously mentioned transcriptomics/proteomics reports cannot discern which mRNAs are translated in axon/synaptic compartments and this information is important since LPS at dendrites and axons is involved in plasticity related processes in the brain (Donlin-Asp et al., 2021), and influence the progression of neurodegenerative diseases (Thelen and Kye, 2019; Lin et al., 2020; Fernandopulle et al., 2021). Moreover, it was recently reported that APP is locally synthetized in synaptosomes of TgCRND8 mice (a model of AD that overexpress the human APP) and that other synaptosome proteins increase its local translation at control animals trained by fear conditioning experiments but not in the AD models (Cefaliello et al., 2020). The authors suggest a synaptic plasticity impairment related to LPS at synapses with an unknown connection with AD development. In the same line the group of Hengst visualized nascent locally synthesized proteins by BONCAT assay in hippocampal neuronal axons exposed to Aβ_1–42_ oligomers and found partial overlap with controls, dysregulation of mRNAs previously reported as AD-related genes including APP, ApoE, Clu, and FERMT2 and novel ones like ATF4 for which was demonstrated its role in neurodegeneration progression (Baleriola et al., 2014). The authors also show an increase on axonal LPS after exposition to Aβ_1__–__42_ and the essential role of Aβ_1__–__42_ for cell death induction. The same group recently reported how translation of axonally localized, but previously silenced sentinel mRNAs, are induced by exposure to Aβ peptide, generating a retrograde signaling complex including vimentin (Walker et al., 2018), and thus, reinforcing the importance of axonal transcriptome and dynamic LPS in disease mechanisms. On the other side, it has been observed that Aβ oligomers can also stimulate tau LPS in the somatodendritic domain mediated by the activation of kinase FYN, using both *in vivo* and *in vitro* models (Li and Götz, 2017). This study proposes a new hypothesis to the somatodendritic accumulation of tau, a common event in AD, although being distance from the axonal or synaptic compartments discussed before.

Despite all these relevant results, the capacity to perform recently developed techniques to evaluate newly synthetized proteins combined with omics approaches including Axon TRAP (Shigeoka et al., 2016; Ostroff et al., 2019), adapted FACS systems (Poulopoulos et al., 2019) or the metabolic labeling protocols (i.e., BONCAT combined with proteomics assays) can be implemented in the near future to compare proteins levels in different neuronal disease models at different developmental stages to identify how LPS can affect neurological disorders, as other suggested (Lin et al., 2020). The later revision provides an interesting meta-analysis indicating that in the top100 expressed transcripts across different axonal transcriptomes and translatomes sets are included genes reported as essential for the development of neuronal disorders.

#### Spinal Muscular Atrophy

Muscle paralysis in motor neuron related diseases, such as Spinal Muscular Atrophy (SMA) and amyotrophic lateral sclerosis (see below), is caused by functional impairment and motor neurons’ degeneration. In 95% of SMA cases an autosomal recessive condition associated with mutations in the survival of motor neurons (SMN1) gene is the cause of the disease. Those mutations reduced but not depleted the levels of full-length SMN protein and this event is sufficient to sustain the survival of most cell types but of spinal motor neurons (Edens et al., 2015).

Disturbed mRNA processing and axonal transport in SMA has been proposed as potential mechanisms leading to the dysfunction and degeneration of motor neurons (Fallini et al., 2012; Chaytow et al., 2018). Interestingly, transcriptome analysis of motor neurons in a mouse SMA model at early presymptomatic stage detects expression-level changes and splicing abnormalities of specific mRNAs critical for motor neuron functions, revealing molecular events that could explain initial key aspects of SMA development (Zhang et al., 2013). Other reports show synaptic related impairments including a reduction in proprioceptive synaptic functions that leads to motor neuron dysfunction, behavior impairments (Fletcher et al., 2017) and the impaired synaptic vesicle release at neuromuscular junction together with postsynaptic side abnormalities in acute SMA stages (Kong et al., 2009; Martínez-Hernández et al., 2013). In addition, it was demonstrated that there are impairments at central synapses during SMA progression, resulting in the loss of excitatory glutamatergic synaptic inputs to motor neurons that may enhance their vulnerability to degeneration and death (Tarabal et al., 2014). All previous results suggest that SMA, typically considered a motor neuron disease, could have dysregulations in other cells, axons, neuronal circuits and central synapses, as suggested previously (Murray et al., 2008; Torres-Benito et al., 2011). Although is out of the scope of this revision, recent reports also suggest that glial cells could have a role in SMA motor neuron degeneration (Abati et al., 2020).

Functional genomics in axons and synapses could shed light in the understanding of SMA pathology, especially at early stages. Nevertheless, those approaches are not abundant in the literature. In this regard, Sendtner’s group analyzed axonal transcriptome changes by microarray analysis in neurons under-expressing Survivor Motor Neuron (SMN) mRNA, one of the deficient proteins in SMA (Saal et al., 2014). In SMN knock down, mRNA levels were affected in somatodendritic and axonal compartments and a great proportion of axonal mRNAs were downregulated compared to axons in basal conditions with gene ontologies related to synaptic localization, neuron projections and growth cones suggesting defects in axon elongation and presynaptic differentiation.

A recent publication analyzed the proteomics on isolated synaptosomes of spinal cords synapses of P14 Smn2B/- mice, a model of SMA (Eshraghi et al., 2019). The authors report 2,030 proteins from which 65 proteins are differentially expressed in relation to the controls at the early presymptomatic stages with enriched molecular functions related to mitochondrial dynamics, cholesterol biogenesis, and protein clearance. This small group of proteins could lead to neurotoxicity dependent cell death and synaptic dysfunction that can be further studied to discern their role in potential therapies that bring stability to the central synapses and preserve the neuronal integrity.

#### Amyotrophic Lateral Sclerosis

Amyotrophic Lateral Sclerosis (ALS) is a lethal motor neuron disease with no current treatment, which is characterized by progressive loss of upper and lower motor neurons at the spinal or bulbar level. The long axons of these neurons become damaged at the initial stages of ALS and the axonal dysfunction appears prior to the motor phenotype of the disease (Fischer et al., 2004; Roy et al., 2005), suggesting that ALS could be redefined as a distal axonopathy disease (Moloney et al., 2014). Recent studies reveal that the axo-pathomechanisms in ALS include problems in neuronal cytoskeleton, cargo transport, axonal energy supply, clearance of junk proteins, and aberrant axonal branching [reviewed by Suzuki et al. (2020)]. Mutations in SOD1, C9orf72, TARDBP, and FUS are known genes contributing toward ALS (Rosen, 1993; Kwiatkowski Jr., Bosco et al., 2009; Vance et al., 2009; DeJesus-Hernandez et al., 2011).

Other authors propose that ALS can be understood as a synaptopathy due to perturbations of synaptic structure and function, glutamate excitotoxicity, impaired transport defects, and free radical-mediated oxidative stress [reviewed by Zarei et al. (2015)] at early stages of the disease before the appearance of patient symptoms due to loss of motor neurons [reviewed by Fogarty (2019)]. In this context, different reports show how the dysregulation of synaptic related elements can trigger ALS development. For example, the poly-glycine-alanine aggregates cause a reduction in SV2 protein synaptic release leading to neuronal death that can be rescued by restoring SV2 levels, thus opening a possible early clinical ALS therapy (Jensen et al., 2020). The mitochondrial dysfunction at the synaptic clefts of ventral horns was also proposed as a possible trigger of sporadic ALS (Engelen-Lee et al., 2017). The overexpression of TDP-43 mutants reduce excitability within pyramidal neurons and attenuates synaptic function associated with ALS progression (Jiang et al., 2019). Previous reports clearly show how ALS is a neuronal disorder in which subcellular local aspects must be considered, including axonopathy and synaptopathy related studies by functional omics (Casas et al., 2016).

Regarding axonopathy related studies, Rotem et al. (2017) analyzed the axonal and somatic transcriptomes of mice with SOD1^*G93A*^ and TDP43^*A315T*^ ALS mutations and found that the genes enriched in axons of both SOD1^*G93A*^ and TDP43^*A315T*^ mutations were associated with mitochondrial function, as previously reported in several ALS models. However, most of the altered genes in the SOD1^*G93A*^ mutation model were not altered in the TDP43 system, and vice versa. This result is consistent with the ALS pathology behavior, which is indistinguishable between the ALS SOD1 and the ALS TDP43 phenotypes. In addition to the mRNA analysis, the authors also describe a list of several miRNAs which have been proposed as potential ALS biomarkers and could be contributors to disease progression (Foggin et al., 2019; Joilin et al., 2020).

Hedlund group analyzed the axonal transcriptomes in ALS models performing an in-house method called “Axon-seq” in cultured mice and human mESC derived motor neurons with the SOD1^*G93A*^ mutation (Nijssen et al., 2018). This method combines compartmentalized microfluidic devices, RNA-Seq and specific bioinformatic analysis. The authors detect up to 5,000 mRNAs in mouse and human stem cell-derived motor neuron axons with oxidative energy and ribosome production as the top identified molecular functions. In relation to ALS, they identified 121 dysregulated genes in neurons containing the SOD1^*G93A*^ mutation and only two of these genes, Zfand1 and Zfp688, were also dysregulated in the SOD1^*G93A*^ soma compartment, suggesting that axon specific changes might occur during disease progression.

Although the previous two groups employed the same mutation model for ALS, they analyzed transcriptomes from different neuron types (spinal cord cultured neurons and mESC derived motor neurons) and used different axon isolation protocols. This might explain why the results between these experiments are barely comparable, as they detect a very different number of total axonal transcripts. While in Rotem et al. (2017) the authors detected more than 15,000 mRNAs localized in axons, Nijssen et al. (2018) detected less than a half of the mRNAs (4,479 with RPKM > 1 or 6,568 with RPKM > 0.1). Nijssen et al. (2018) argue that their sequencing method (Axon-seq) detected less mRNAs because previously reported methods may have cross-contamination between somatodendritic and axonal compartments. The lists of differentially expressed genes in SOD^*G93A*^ for both studies have no overlapping, denoting how different experimental procedures and patient backgrounds can affect the results obtained.

In the case of Aoki’s group study, they performed RNA profiling of isolated axons from nerve organoids which mimics motor nerve tissue (Akiyama et al., 2019). Using genetic tools, the authors produce isogenic hiPSCs-derived motor neurons carrying the single aminoacid mutation p.H517D in the FUS gene. After RNA-Seq analysis, the authors detected 671 differentially expressed genes in the axon compartment compared to the cell bodies. From this list they highlighted Fos-b, the binding target of FUS, with increased levels in FUS mutant motor neurons and conclude that this factor could be a key regulator of FUS-mutant axon branching *in vitro* and *in vivo* suggesting that Fos-B pathway could be a potential therapeutic target in future ALS research.

Regarding synaptic related omics, synaptosome extraction from SOD1^*G93A*^ and wild type mice combined with a dynein immunoprecipitation step and mass spectrometry was used to identify disrupted interaction between this retrograde motor protein and Staufen1, an essential RNA binding protein for transport and localization of neuronal RNAs in axon and synapses (Gershoni-Emek et al., 2016). The authors suggest that this disrupted interaction could lead to synaptic dysfunction and motor neuron toxicity in ALS.

## Discussion

The application of functional genomics approaches has contributed to our understanding of the molecular characterization of neuronal processes in health and disease. Despite these advances, many questions remain unanswered and there is a clear need to translate these omics findings into detailed mechanistic information of the cellular and physiological processes behind them.

For example, an open question is about the complexity, differences or commonalities in the transcriptome of axons and synaptic fractions isolated from *in vitro* or *in vivo* models. Recent findings reported by us showed that the transcriptome of isolated axoplasm from rat myelinated motor fibers *in vivo* is “less complex” −1,008 mRNAs detected (Farias et al., 2020)- than those reported *in vitro*: 15,555 mRNAs in Minis et al. (2014), 11,145 in Zappulo et al. (2017), 4,553 in Nijssen et al. (2018), and 9,858 in Briese et al. (2016). Although those differences can also be ascribed largely to changes between developmental stages (immature vs. mature), neuronal models and experimental protocols, the results reveal the importance of improving and extending *in vivo* models and patient derived cell systems to better understand neuronal processes. In the case of motor neuron diseases, interesting data could arise from transcriptome studies on axoplasm material isolated from animal models of ALS or SMA. Moreover, the analysis by RNA-Seq of splice variants that can trigger many disease pathologies, and alternative UTR sequences, that regulate the axonally transported RNA populations (Taliaferro et al., 2016; Turner-Bridger et al., 2020), could be an interesting avenue to understand the development of motor diseases (Kar et al., 2018; Tushev et al., 2018; Bae and Miura, 2020). In parallel, important considerations about the contamination of each isolation procedure need to be taken into account since neurites or synaptic components have lower total amounts of RNA/proteins compared to surrounded cell bodies or glial cells. It is expected that current and future studies will rely more on the quality rather than the quantity of starting material. Indeed, single cell and/or spatial transcriptomics with subcellular resolution could contribute toward this problem in the local identification of transcripts minimizing isolation steps. Additionally, we may pose simple but relevant questions: does one type of neuron can have different axonal transcriptomes? Do different neurons have different axonal transcriptome? Is there a core group of mRNAs in the axon or synapses?

Due to the relevance of LPS in axonal development, homeostasis and synaptic plasticity, the identification of locally translated mRNAs could be a step forward in the study of neuronal disease etiology (Kindler and Kreienkamp, 2012; Swanger and Bassell, 2013; Kuzniewska et al., 2020). The use of pulse-chase stable isotopic amino acids, amino acid analogs, or puromycin labeling, coupled with mass spectrometry protein identification from *in vivo* isolated neuronal compartments, could open new avenues to identify the repertoire of newly synthesized proteins at normal or pathological conditions. New protocols, like Axon-TRAP promise to generate pictures of *in vivo* responses in different experimental setups including axonopathies. Performing these protocols in isolated axoplasm material from adult peripheral nerves who carry specific mutations that generate neurological disorders, like ALS, could also be of importance to unravel molecular aspects of these diseases, especially at the early stages.

It is also relevant to consider neuropil activated monosomes from both synaptic compartments (Biever et al., 2020), which together with previous findings on ribosomes assembled at axons independently from the nucleolus (Shigeoka et al., 2019) opens a new window in how to analyze axon/synaptic translatomics. Since monosomes were demonstrated as key contributors to the neuronal translatome, in particular for axonal side regions (Biever et al., 2020), the fact that standard protocols like Ribo-Seq selectively analyze polysome footprints, may provide an incomplete view at least for axonal translational regulation. Moreover, active monosomes in the neuropil seem to prefer longer and complex mRNAs, suggesting that monosome and slow translation could allow post translational modifications that are essential for synaptic plasticity events and for the prevention of protein aggregations, critical in neuronal disorders (Biever et al., 2020). Future analysis in this direction probably will shed light oin how complex disorders like ALS or AD are developed at axon or synaptic levels.

Mass spectrometry technologies have dramatically increased their sensitivity in dynamic and quantitative proteomics. However, analyzing the dynamic changes in the synaptic proteome is a difficult task due to the intrinsic variability in the isolation of the structures of interest (Dieterich and Kreutz, 2016). Moreover, synapses also have a complex architecture with thousands of protein interactions that classical proteomic approaches cannot resolve. The development of chemical cross-linking combined with mass spectrometry techniques, known as XL-MS protocols, allows the capture of both native protein structures and interactions by cross-linking reagents in a physiologically subcellular context of interest (Liu et al., 2015). Recent work applies these protocols to generate the largest cross-linking datasets to date and reveal the complex architecture of the synaptic compartment (Gonzalez-Lozano et al., 2020).

In addition to the challenges derived from the study of synaptic dynamics by proteomics, analytical steps also have to consider the “average problem” (Wang and Savas, 2018), which is produced by highly abundant proteins that can mask the measurement of low abundance but important synaptic proteins. Different protocols like LCM (Kennard et al., 2014), FACS (Biesemann et al., 2014), biorthogonal strategies (Heo et al., 2018), APEX and BioID (de Wit et al., 2009; Uezu et al., 2016) could help to analyze synaptic type-specific proteomes, since they are pseudo-independent of organelle or subdomain purifications (Wang and Savas, 2018).

Another current challenge for synaptic and localized neuronal proteomics is how to study the implicit diversity of the brain connectome. Undoubtedly any precise profiling of RNA and proteins at the different levels of the connectome (cell body, axon, dendrites, and synapse) will make an invaluable contribution to the description of how neuronal networks function, and could facilitate the development of novel therapeutics for neurological disorders in the future. Nevertheless, initial metadata analysis of synaptic neuroproteomics of independent experiments developed in the same neurological disorder’s models reveal that only few detected proteins are constantly dysregulated (Reig-Viader et al., 2018) suggesting that a more standardized and systematic research is needed to identify the key pathophysiological mechanisms involved. Future research in the field may require larger-scale experiments combining proteomics and biochemical validations in order to address the intrinsic methodological and biological variability.

Synaptic proteomics faces additional challenges in the study of post-translational modifications, since plasticity-related processes like Long Term Potentiation initiation requires SUMOylation (Daniel et al., 2018) and phosphorylation of many pre- and postsynaptic proteins (Lee, 2006; Yokoi et al., 2012; Li et al., 2016). The post-translational modifications increase peptide spectra and consequently reducing the depth of traditional proteomic analyses that are being approached using recent preparation approaches and MS search algorithms, like open search (Chick et al., 2015).

A complementary approach to standard functional genomics is the characterization of mRNA composition by spatial transcriptomics (see Section “Transcriptomics and Translatomics at the Synaptic Compartments”). These approaches provide an unprecedented resolution (Luecken and Theis, 2019) and could serve as a bridge between neuroscience, computational biology, and systems biology, enabling a better understanding of the brain’s cellular heterogeneity and its subcompartment complexity too. In combination with powerful omics and expansion microscopy (i.e., ExSeq) there are promising protocols to analyze difficult to isolate compartments and bring new insights about the RNAs present in specific neuronal compartments at basal or neuronal disease conditions (Kulkarni et al., 2019).

As many new routes of research lay in the future, it remains to be investigated the local role of other regulatory components by omics approaches like non-coding RNAs in axons (Natera-Naranjo et al., 2010; Sasaki et al., 2014; Corradi and Baudet, 2020) as well as synapses (Earls et al., 2014; Smalheiser, 2014; Epple et al., 2021), or different regulatory pathways controlling local protein synthesis impacting local physiology of an individual axon or synaptic button (Terenzio et al., 2018). In summary, the dynamic quantification of transcripts and proteins localized in each neuronal compartment by functional genomics, in combination with sophisticated methods for isolation/visualization of the biological material, will help to understand the molecular changes occurring in these cytoplasmic territories in basal conditions and their involvement in neuronal disorders.

## Author Contributions

JRSS designed and planned the revision contents. AD and JRSS wrote the manuscript and designed the figures. AD and JG developed the tables and searched for references. JG, GE, JF, FDB, and PS, contributed to specific sections according to their expertise. AD and JRSS prepared the final corrected version. All authors contributed to the proofing, corrections and approved the final version of the article.

## Conflict of Interest

The authors declare that the research was conducted in the absence of any commercial or financial relationships that could be construed as a potential conflict of interest.
